# The Nexus between Bovine Tuberculosis and Fasciolosis Infections in Cattle of the Kafue Basin Ecosystem in Zambia: Implications on Abattoir Surveillance

**DOI:** 10.1155/2012/921869

**Published:** 2012-11-10

**Authors:** Musso Munyeme, Hetron Mweemba Munang'andu, Andrew Nambota, John Bwalya Muma, Andrew Malata Phiri, King Shimumbo Nalubamba

**Affiliations:** ^1^Department of Disease Control, School of Veterinary Medicine, University of Zambia, P.O. Box 32379, Lusaka 10101, Zambia; ^2^Section of Aquatic Medicine and Nutrition, Department of Basic Sciences and Aquatic Medicine, Norwegian School of Veterinary Sciences, P.O. Box 8146, 0033 Oslo, Norway; ^3^Department of Clinical Studies, School of Veterinary Medicine, University of Zambia, P.O. Box 32379, Lusaka 10101, Zambia

## Abstract

Bovine tuberculosis (bTB) and fasciolosis are important but neglected diseases that result in chronic infections in cattle. However, in Zambia, these diseases are mainly diagnosed at abattoirs during routine meat inspection. Albeit the coinfection status, these diseases have been reported as nothing more than normal separate findings without an explanatory phenomena. Forthwith, we formulated this study to assess the possible association of the two diseases in a known high prevalence area on the Kafue basin ecosystem. Of the 1,680 animals screened, 600 (35.7%; 95% CI 33.4%–38%) and 124 (7.4%; 95% CI 6.1%–8.6%) had fasciolosis and tuberculous lesions; respectively, whilst 72 had both fasciola and tuberculous lesions representing 12% (95% CI 9.4%–14.6%) and 58.1% (95% CI; 49.3%–66.7%) of the total positives for fasciola and tuberculosis, respectively. Jaundice was seen in 304 animals, 18.1% (95% CI; 16.3%–19.9%) and was significantly correlated to fasciolosis (*r* = 0.59, *P* < 0.0001). A significant association (*χ* ^2^ = 76.2, df = 1, and *P* < 0.0001) was found between fasciolosis and tuberculous lesions. Simple logistic regression intimated fasciolosis as a strong predictor for tuberculous lesions with animals that had fasciola being five times more likely to have tuberculous lesions (odds ratio = 4.8, 95% CI: 3.3–7.0). This study indicates that transmission and spatial risk factors of communicable and noncommunicable diseases such as bTB and fasciolosis can be correlated in an ecosystem such as the Kafue flats.

## 1. Introduction 

In Zambia, bovine tuberculosis (bTB), caused by *Mycobacterium bovis*, and fasciolosis caused by *Fasciola gigantica* have been reported to be causing considerable economic losses to the livestock industry annually through abattoir condemnations accompanied with loss of production efficiency [1–9]. The frequency of abattoir condemnations from the two conditions has been well documented in Zambia through various studies that have shown direct losses to the general agribusiness and the livestock industry in particular [2, 5, 7]. Whereas bovine tuberculosis (bTB) is one of the most intractable diseases of cattle in Zambia [3], fasciolosis on the other hand is treatable despite its high prevalence and endemicity, mainly due to neglect [7]. These disease conditions have always been considered separately despite showing a concurrent infection in nature only apparent through abattoir findings. 

Infection by the parasitic trematode *Fasciola gigantica *is acquired by cattle through the ingestion of vegetation on which the infective metacercariae have encysted. The metacercariae excyst in the intestine, burrow through the gut wall of the mammalian host, and migrate across the body cavity to the liver, where the parasite causes extensive damage resulting in elevated immunoglobulin E levels, eosinophilia, and immune responses associated with the Th2 subtype [10, 11]. This type of immune response has been shown to suppress *Mycobacterium bovis*-specific Th1 response and consequently causes a delayed bacterial clearance from the affected lungs coinfected with *F. gagintica* [10, 11]. In contrast, *Mycobacterium bovis *infection has been shown to have no effect on the *F. gigantica*-specific Th2 response or on liver pathology [12]. This immunomodulatory effect suggest that fasciolosis infection can enhance tuberculosis infection in susceptible hosts. On the contrary, tuberculosis infection has been shown not to enhance fasciolosis infection [10]. Other immunological studies have shown that concurrent infections with chronic parasitic infections assist in the downregulation of the animal's immune system resulting in increased susceptibility to infection [13]. Thus, in terms of gross pathological lesions of fasciolosis detected during meat inspection, and given the pathobiology and immune response of both diseases, a correspondingly close inspection for tuberculous lesions must be instituted [14]. However, further detailed studies need to encompass the environmental aspects of disease transmission. Whereas fasciolosis is a noncommunicable disease more linked to the environment, tuberculosis on the other hand is a communicable disease associated with close contact between infected and susceptible animals and to a lesser degree the environment. Although both disease conditions can be transmitted through a similar route, oralfecal, compared to fasciolosis, tuberculosis has been shown to depend more on the respiratory route. However, in the lack of detailed diagnostic and controlled studies, concurrency in the infection of cattle by fasciolosis and tuberculosis needs careful interpretation. 

Currently, Zambia remains one of the countries with a low cattle density in the subregion despite having a grazing area of 20.3 million hectares (ha) that supports a paltry 3 million cattle compared to Zimbabwe's 12.1 million ha which supports 5.4 million cattle [15]. Despite all three of Zambia's agroecological zones being suitable for cattle production, only the Kafue and Zambezi ecosystems support approximately two-thirds of the national cattle herd [15]. In these same ecosystems, abattoir surveys have shown a rising problem of bTB and fasciolosis where both disease conditions appear to be endemic [2, 7, 9]. Lack of a trace back system for disease control purposes poses a great challenge to the development of the traditional livestock industry which accounts for over 80% of cattle production in Zambia [15]. Under this very sector, cattle are reared under natural and open populations depending on and sharing of common pool resources (CPRs) such as grazing grounds and watering points, which constitutes a major risk factor for disease transmission such as bovine tuberculosis [16–19]. Under this sector, chronic livestock disease conditions have been neglected to a level that even those diseases that are treatable like fasciolosis, little or no mitigatory measures are applied [5]. The aim of the present study was to assess the correlation between BTB and fasciola infections through meat inspection and use that information to evaluate its potential implication on abattoir surveillance.

## 2. Methods 

### 2.1. Study Area

**Figure 1:**
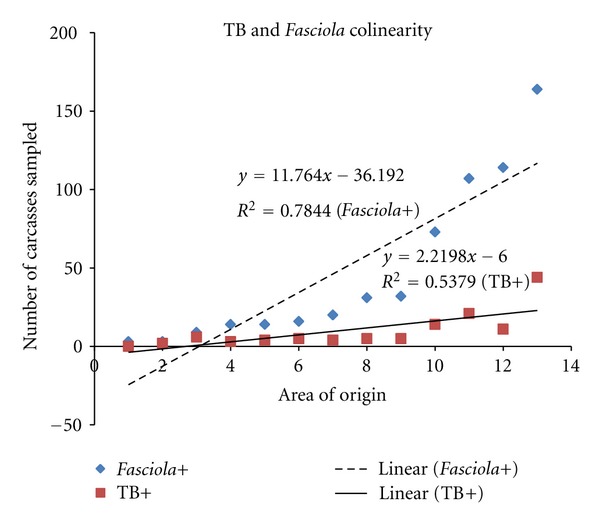
Map showing study area.

The study was carried out at the abattoirs located in the Namwala district, where almost the entire cattle population is reared under the traditional system and as such routine tuberculosis testing or deworming of animals is rarely practiced. Namwala is one of the districts in the country with the highest cattle population making it the major supplier of beef to other areas in the country (Figure 1) [2]. The district has an organized system of cattle rearing clubs despite lack of disease intervention measures (Figure 1). The location of the district right within the Kafue flats makes it the most suitable for sampling [2, 5–8]. The climate is tropical, covering two main seasons: the dry season covering the period between April to October and the wet season from November to March with minimum and maximum temperatures ranging from 19 to 36°C in October and 0 to 21°C in July. Annual rainfall averages between 1100 mm and 1200 mm leading to flooding during the rainy season. The river originates from upstream areas that receive much higher annual rainfall in the north (approximately 1,400 mm) and contributes to the major part of the flooding leading to inundating of large areas within the basin. 

### 2.2. Data Collection

A survey of 1,680 cattle presented for slaughter at Namwala abattoir was undertaken to assess the correlation of fasciola and bTB. During this time, information of owners, origin of the animals, sex, age, herd size, disease history, and other relevant data were recorded. In most cases, the traders who brought the animals had no data on herd sizes, disease history, and age. This meant that very little information was collected on these aspects. In some instances, animals were brought to the abattoirs by middle men from Namwala central, who may have bought the animals from farmers without taking note of their origin. All such animals were categorized under Namwala central. Postmortem inspection was conducted as described elsewhere [20], and inspection procedures included visual examination of organs and lymph node, palpation, and incisions being made where necessary. 

### 2.3. Case Definitions

Meat inspection was done by local meat inspectors at the abattoir who had received prior in-house training on how to conduct detailed postmortem inspections and on how to capture necessarily data. Organs suspected of having fasciolosis and tuberculosis lesions were collected and marked as such. 

#### 2.3.1. Examining for Tuberculosis Lesions

From all slaughtered cattle-detailed post-mortem examinations were conducted with particular attention to the lymph nodes for visible lesions characteristic of tuberculosis: granulomas with or without caseous necrosis and mineralization. Particular lymph nodes that were examined from each animal (both right and left lymph nodes, where they were present bilaterally) included parotid; mandibular; lateral and medial retropharyngeal; superficial cervical; cranial, middle, and caudal deep cervical; tracheobronchial; cranial, middle, and caudal mediastinal; hepatic; mesenteric; deep popliteal; subiliac; medial iliac; and supramammary. In addition all visceral organs starting with the lung, liver, spleen, kidney, and mammary glands were examined for characteristic granulomatous lesions (with central caseous necrosis and mineralization). The entire carcass was also carefully examined for gross lesions of tuberculosis. The findings of tuberculous lesions in any of the examined organs meant that the carcass was positive for tuberculosis based on gross post-mortem examination. 

#### 2.3.2. Acid Fast Staining and Culturing

All the TB suspect tissue and organs were decontaminated at the laboratory. Fat was trimmed off from the suspect material of which some 3 to 6 g was measured in a sterile mortar to which 10 mL of 4% sodium hydroxide (NaOH) was added. An equal amount of sterile sand was added to the contents in the mortar which were then ground. The products were then transferred to McCartney bottles and centrifuged at low speed of 3000 rpm for 10 minutes. After which the supernatant fluid was decanted off, and 20 mL of sterile H_2_O was added to the sediment and mixed vigorously by shaking on a vortex to a uniform homogenate. The contents were again centrifuged at low speed of 3000 rpm for 20 minutes, and the supernatant fluid was decanted. The sediments of these decontaminated homogenates were inoculated in duplicate Lowenstein-Jensen media slants containing glycerol and 0.4% sodium pyruvate to enhance the isolation of *M. bovis* and incubated aerobically at 37°C for 8 weeks, with twice weekly observations for growth and colony characteristics. The resulting cultures were tentatively identified as probable *Mycobacterium tuberculosis* complex by their slow growth and colony characteristics. Purity and acid fastness of the colonies were checked through the Ziehl-Neelsen staining. 

#### 2.3.3. Examining for Fasciola Lesions

For fasciolosis, the liver was examined in detail for visible lesions suggestive of infection. The appearance of hypertrophied of the bile ducts with actual adult liver flukes and sometimes accompanied with frank mechanical obstruction of the biliary ducts was taken as a positive case. This was assisted by making several incisions on the liver apart from palpation and observation. 

### 2.4. Data Storage and Statistical Analysis

The database was established in Microsoft excel spreadsheets before transferring it to Stata SE/10 for Windows (Stata Corp. College Station, TX, USA). Proportion estimates for fasciolosis and tuberculosis positives with confidence intervals were computed using the survey command estimates in Stata with adjustments for strata (area of origin) as described by Dohoo and coworkers [21]. Chi-square was used to determine the levels of association between fasciolosis and tuberculosis at the abattoir. Further, data was analyzed according to area, and correlations between fasciola and tuberculosis infection were analyzed for each strata/area of origin. Simple logistic regression was used to analyze the relationship between fasciolosis and tuberculosis with linear relationship being determined through simple linear regression as described by Dohoo and co-workers [21]. 

## 3. Results 

### 3.1. Univariate Analysis

Out of 1,680 animals screened, 1,479 (88%) were male animals with 201 (12%) being female animals. Of the total screened, 600 (35.7%; 95% CI 33.4%–38%) had lesions suggestive of fasciola whilst 124 (7.4%; 95% CI 6.1%–8.6%) had lesions suggestive of tuberculosis. 

Of the 600 animals that were positive for fasciola on post-mortem, 72 animals [12% (95% CI 9.4%–14.6%)] had lesions suggestive of tuberculosis, while of the 124 animals that were positive for tuberculosis, 72 [58.1% (95% CI; 49.3%–66.7%)] had lesions suggestive of fasciola infection. From all the animals sampled, jaundice was seen in 304 animals [18.1% (95% CI; 16.3%–19.9%)] and was found to be significantly correlated to fasciolosis (*r* = 0.59, *P* < 0.0001). 

### 3.2. Acid Fast Staining and Culturing Results

Of the 124 tissue homogenates that were tested for acid fast bacilli organisms, only 53 [42.7% (95% CI 19.5%–47.3%)] showed positive staining characteristics of acid fast organisms. On culture, 67 samples had positive growths [54% (95% CI 49.6%–61.8%)].

### 3.3. Associations and Regression Analysis

**Figure 2:**
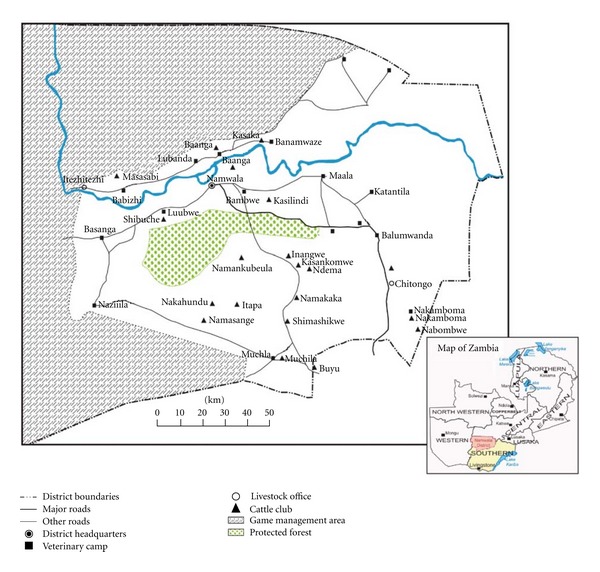
The graph indicates correlation of tuberculosis and fasciola condemned organs to area of origin. Note that at slaughter, animals with high fluke condemnation rate corresponded to those for tuberculosis by area of origin.

Data was analyzed according to area, and correlation between fasciola and tuberculosis infection was analyzed for each area (Figure 2 and Table 1). Out of the 13 areas that supplied animals to the abattoir during the sampling period, 12 areas showed a positive correlation between fasciola and tuberculosis infection with only one area showing a weak and negative correlation. There was a statistically significant relationship between fasciolosis and tuberculosis positive animals (*χ* ^2^ = 76.2, df = 1, *P* < 0.0001). Animals that were found to be jaundiced on post-mortem examination were significantly associated with fasciola infections (*χ* ^2^ = 595, df = 1, *P* < 0.0001). 

When the linear relationship between fasciolosis and tuberculosis was further determined by simple linear regression, it was evident that the interaction of tuberculosis and fasciolosis was positive (coefficient = 0.3, *t* value = 8.93, and *P* < 0.0001). On simple logistic regression, fasciolosis was a strong predictor of tuberculosis as animals that were found to be condemned of fasciola were nearly five times more likely to have tuberculosis (odds ratio = 4.8, 187 95% CI: 3.3–7.0). Figure 2 shows that animals with a high fluke condemnation rate corresponded to those condemned for tuberculous lesions by area of origin. 

## 4. Discussion 

Abattoir surveillance provides vital information with regards to diseases with an insidious onset and those which are pathogenic yet having a low virulence. Such diseases may not present overt clinical signs early enough to warrant timely detection for mitigation purposes hence the abattoir being a vital screening point. In short, abattoir surveillance may be regarded as a bridge between laboratory and field based diagnostics, particularly in resource poor countries such as Zambia. However, careful interpretation of abattoir results is needed given its limitations such as low sensitivity of the diagnostic test used based on post-mortem examination [22]. However, this did not affect the interpretation of results considering that no test is 100% sensitive or 100% specific [22]. The lack of control over events, that is, during the study period we discovered that it is difficult to obtain all the information needed owing to the middle men who are cattle buyers. 

Despite limitations linked to abattoir syndromic surveillance, this study has been able to demonstrate that fasciolosis and tuberculosis infections are positively correlated. Further, within its limits, this study has been able to indicate the likelihood of synergistic effects of fasciolosis on the susceptibility and possible progression of tuberculosis in cattle. This finding has major implications on the way surveillance; diagnostics and traditional management practices are conducted where disease conditions are considered as separate entities without considering the potential impact of concurrent or multiple infections. However, disease interactions in nature may be dependent and even fuelled by other infections and factors. However, it is difficult to empirically demonstrate this assertion. Our findings of a correlation between fasciolosis and tuberculosis in cattle warrant further investigation given that our current study was based on observations of results on events that took place in natural open populations. Experimental studies may help in understanding the biological plausibility and pathobiology of both diseases taking into account their mechanisms of interaction in relation to the host immune response. 

Due to missing data (>30%) on age and body condition scores, it was difficult to analyse and relate emaciation, age, and jaundice observed in some of the carcasses that had their livers condemned to be due to bTB and fasciolosis. However, in those cases where data was present, we were able to demonstrate that jaundice was significantly correlated to fasciolosis. Although not conclusively proven, however, these results intimate synergism between fasciolosis and tuberculosis in facilitating the latter's susceptibility given the chronic nature of both diseases. Nonetheless, because of the design of our study, it was not possible to prove a cause and effect of each of the disease entities. Similarly, it was not possible to determine which disease infected the host first or that there was simultaneous infection, and the sequence of subsequent pathological processes.

Parasitic infections have been shown to downregulate cell-mediated immunity which is required to contain the invading tuberculosis bacteria [23, 24]. Further, it is known that fasciolosis may suppress the host animal's cellular response to diseases, which is the main type of immunological response towards tuberculosis infection [24, 25]. Studies by Brady and co-workers have been able to demonstrate that Fasciola infections suppress Th1 responses against bacterial infection [11, 23]. They achieved this through evoking Th2 and inducing a bystander suppression of protective Th1 responses to bacteria with the mediation of IL-4 as a modulator [11, 23]. The abrogation of this type of immune response in a susceptible host may mean increased risk particularly to tuberculosis infection which depends mainly on macrophage activation during infection, which is also a similar type of response evoked by helminthes infection [25]. This phenomenon was supported by our finding that showed that fasciolosis is a strong predictor of tuberculosis occurrence in cattle. 

We further argue that the biological mechanisms involved in disease transmission and coinfection of bTB and fasciolosis need to be fully addressed. Further needs to be done on underlying factors responsible for the observations made during routine meat inspection and based on natural occurring disease phenomena. 

## 5. Conclusion 

Our findings in this study highlight the disease interaction and concurrent nature of infections open free-living livestock populations. To a greater degree, these results have a very important practical application to countries facing a high burden of livestock diseases to assess their concurrency in infection in relation to their pathobiology. Remotely, our study suggests the possibility of ecological involvement in the dynamics of infectious diseases within individual hosts as a function of a complex interplay between host immunological factors and disease distribution. 

## Figures and Tables

**Table 1:** Proportion estimates and linear correlation of tuberculosis and fasciolosis positives based on abattoir survey by area of origin (*n* = 1,680, February to June of 2008) in Zambia.

Origin of	No. supplied	Abattoir Positives	95% CI	Condemned livers^¥^	95% CI	Correlation TB and
animals		TB (%)	(%)	(%)	(%)	fasciola by area (*r*)
Namwala^€^	294	21 (7.14)	(4.2, 10.1)	107 (36.39)	(30.9, 41.9)	0.26
Maala	499	44 (8.82)	(6.3, 11.3)	164 (32.87)	(28.7, 36.9)	0.20
Basanga*	193	14 (7.25)	(3.5, 10.9)	73 (37.82)	(30.1, 44.7)	0.15
Lubwe*	308	11 (3.57)	(1.4, 5.6)	114 (37.01)	(31.6, 42.4)	0.14
Bambwe	50	4 (8.0)	(0.4, 15.6)	20 (40.00)	(26.2, 53.7)	0.30
Katantila*	7	0 (0.0)		3 (42.86)	(3.2, 82.4)	
Namusonde	31	3 (9.68)	(0.0, 20.3)	14 (45.16)	(27.0, 62.9)	0.15
K/mwanda	13	2 (15.38)	(0.0, 35.8)	3 (23.08)	(0.0, 46.9)	−0.12
Banamwaze*	56	5 (8.93)	(1.3, 16.5)	16 (28.57)	(16.6, 40.5)	0.12
Kantengwa*	65	5 (7.69)	(1.2, 14.2)	32 (49.23)	(36.9, 61.4)	0.50
Chitongo*	84	5 (5.95)	(0.1, 11.0)	31 (36.90)	(26.5, 47.2)	0.31
Muchila*	42	6 (14.29)	(3.5, 25.0)	9 (21.43)	(8.9, 33.9)	0.55
Itezhi-tezhi*	38	4 (10.53)	(0.1, 20.4)	14 (36.84)	(21.0, 52.3)	0.01

Overall	1,680	24 (7.38)	(6.1, 8.6)	600 (35.7)	(33.4, 38.0)	0.13

Some cattle owners were identified by their villages, and these were then allocated to the nearest block area operated by a veterinary camp for ease of stratifying the data by area (refer to Figure 1). ^¥^Only whole liver condemnations were considered as condemnations, not partial trimmings, and only jaundice was included and no other lesions that may explain presence of chronic fasciolosis. ^€^For Namwala Central, some were actual owners of the animals whilst a greater majority were cattle traders, despite animals being bought somewhere else, which were recorded under Namwala Central due to lack of recall of places of origin.
